# A Simple Algorithm for Averaging Spike Trains

**DOI:** 10.1186/2190-8567-3-3

**Published:** 2013-02-25

**Authors:** Hannah Julienne, Conor Houghton

**Affiliations:** 1 School of Mathematics, Trinity College Dublin, Dublin, Ireland; 2 School of Physiology & Pharmacology, University of Bristol, Medical Sciences Building, University Walk, Bristol, BS8 1TD, England; 3 Department of Computer Science, University of Bristol, Merchant Venturers Building, Woodland Road, Bristol, BS8 1TD, England

## Abstract

Although spike trains are the principal channel of communication between neurons, a single stimulus will elicit different spike trains from trial to trial. This variability, in both spike timings and spike number can obscure the temporal structure of spike trains and often means that computations need to be run on numerous spike trains in order to extract features common across all the responses to a particular stimulus. This can increase the computational burden and obscure analytical results. As a consequence, it is useful to consider how to calculate a central spike train that summarizes a set of trials. Indeed, averaging responses over trials is routine for other signal types. Here, a simple method for finding a central spike train is described. The spike trains are first mapped to functions, these functions are averaged, and a greedy algorithm is then used to map the average function back to a spike train. The central spike trains are tested for a large data set. Their performance on a classification-based test is considerably better than the performance of the medoid spike trains.

## 1 Introduction

Spike trains are highly variable, with the same stimulus causing different responses for different trials. While a stimulus will modulate a neuron’s firing pattern on a longer timescale, noise will affect spike timings on a shorter timescale, masking the message encoded [3]. Therefore, it would often be useful to be able to summarize a set of such responses by averaging them, giving a single exemplar. This would speed up computations based on spiking responses and focus studies of coding in spike trains on features that are common across all responses to a given stimulus. 

There have been numerous attempts to effectively summarize responses. These include the calculation of the peristimulus time histogram or spike density function [4,5,20] and the development of algorithms to calculate an ‘average’ or ‘central’ spike train [29]. Here, an algorithm for averaging spiking responses is proposed. It uses an average filtered function to construct a central spike train. The spike trains are mapped into the space of functions by filtering them with a causal exponential filter. The average of these functions is calculated. This average function is then mapped back to a spike train by finding a sequence of spikes whose filtered function is close to the average function. 

This is an instance of the well-studied problem in kernel methods of finding the pre-image of a point [18]. Here, the calculation is performed approximately using a greedy algorithm, a type of matching pursuit algorithm [13]. It can be implemented efficiently in this case because of the exponential filter used to map the spike trains to the space of functions. 

These central spike trains are tested on a large data set recorded from zebra finch auditory neurons by the Frederic Theunissen laboratory and made available on the Collaborative Research in Computational Neuroscience database [1]. The effectiveness of the central spike train in summarizing the set of responses is studied in various ways. Perhaps most importantly, the central spike trains are tested using a transmitted information measure of metric-based classification. The performance of the central spike train as a classification template is compared to the performance of the obvious alternative, the medoid response. The medoid of a set of responses is taken to be the response in the set with the lowest average distance from the rest of the set. It is found that the central spike trains appear to be considerably more effective at summarizing the responses than the medoid responses. 

## 2 Methods

The algorithm works by mapping all the spike trains to functions, averaging these in the function space and then finding the spike train that best corresponds to this average function.

The first part of the algorithm is the map from spike trains to functions, which is done by filtering. Given a spike train

(1) $$u=\{u_{1},u_{2},\ldots ,u_{m}\}$$

filtering maps it to a real function, f(t;u), using a kernel k(t):

(2) $$u\mapsto f(t;u)=\sum_{i=1}^{m}k(t-u_{i}).$$

The kernel function has to be specified. Here, the causal exponential is used

(3) $$k(t)=\{\begin{array}{cc} 0 & t<0\\ \sqrt{\frac{2}{\tau }}e^{-t/\tau } & t\geq 0 \end{array}$$

where the normalization factor of $\sqrt{2/\tau }$ is convenient because it means $\int_{-\infty }^{\infty }k{\left(t\right)}^{2}\;dt=1$. *τ* is a timescale. In the example considered in the Results section (Sect. 3), this timescale is chosen to match the timescale associated with the optimal metric-based clustering of the responses.

The choice of kernel function is motivated by the van Rossum metric, which also involves filtering [24]. Indeed, the whole approach is motivated by the idea, illustrated by the van Rossum metric that a useful way to calculate with spike trains is to first map them into the space of functions. This is in the spirit of the kernel-based smoothing often performed in estimating inhomogeneous neuronal firing rates and also in the spirit of the reproducing kernel Hilbert space framework for spike train signal processing described in [15]. 

Now, given a collection of spike trains

(4) $$\left\{u_{1},u_{2},\ldots ,u_{n}\right\}$$

the filtering gives a collection of functions

(5) $$\left\{f(t;u_{1}),f(t;u_{2}),\ldots ,f(t;u_{n})\right\}$$

and these are averaged to give

(6) $$\bar{f}(t)=\frac{1}{n}\sum_{a=1}^{n}f(t;u_{a}).$$

The central spike train is then the spike train $\bar{u}$ that filtering maps closest to the function average $\bar{f}(t)$. Here, closest is defined using a square error, so the central spike train $\bar{u}$ minimizes

(7) $$E(\bar{u})=\int {\left[\bar{f}(t)-f(t;\bar{u})\right]}^{2}\;dt.$$

**Fig. 1:**
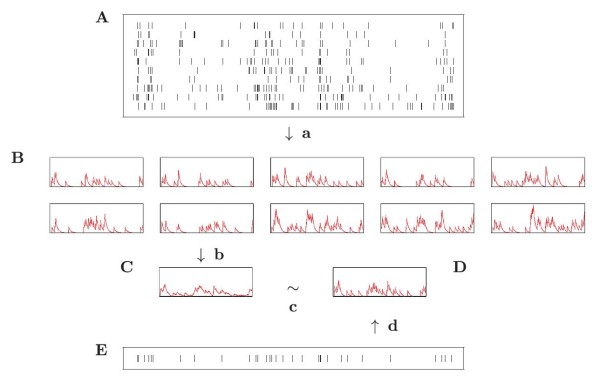
A schematic representation of the averaging algorithm. **A** is a raster of spiking responses to a single stimulus. These are converted to functions by filtering (**a**). **B** shows the collection of functions that results. These are averaged (**b**) giving the average function **C**. This average function is approximated (**c**) by **D**, a function which itself is the result of filtering. This filtering is represented by **d** and the corresponding spike train by **E**. The optimal choice of **E** is the central spike train $\bar{u}$. The greedy algorithm is used to estimate this

This is illustrated in Fig. 1. However, rather than trying to solve this difficult minimization problem exactly, a greedy algorithm is used as an approximate approach. 

In the greedy algorithm, spikes are added to the central spike train $\bar{u}$ one-by-one, with each successive spike time chosen to reduce the remaining error as much as possible. Thus, at an intermediate point, some spikes have already been added to the central spike train $\left\{{\bar{u}}_{1},{\bar{u}}_{2},\ldots ,{\bar{u}}_{p-1}\right\}$ and the location of the *p*th time ${\bar{u}}_{p}$ needs to be decided. It is chosen to minimize

(8) $$\begin{array}{rcl} \delta E({\bar{u}}_{p}) & = & E({\bar{u}}_{1},{\bar{u}}_{2},\ldots ,{\bar{u}}_{p-1},{\bar{u}}_{p})\\ & & -E({\bar{u}}_{1},\bar{u_{2}},\ldots ,{\bar{u}}_{p-1}) \end{array}$$

where δE is negative if the new spike time lowers the error. In fact, for the causal exponential kernel, it is easy to calculate δE analytically. Integrating gives

(9) $$\delta E({\bar{u}}_{p})=1+2\sum_{j=1}^{p-1}e^{-|{\bar{u}}_{j}-{\bar{u}}_{p}|/\tau }-\frac{2}{n}\sum_{a,i}e^{-|u_{ai}-{\bar{u}}_{p}|/\tau }.$$

The value of this function is easily computed and so it can be rapidly minimized with respect to ${\bar{u}}_{p}$ using, for example, the Brent or golden section method [17]. 

It might seem that the algorithm should continue only while the minimum value of δE is negative. However, this tends to give central spike trains with fewer spikes than the average spike number in the collection. This appears to be an artefact of the use of a causal filter. Roughly speaking, if a spike time ${\bar{u}}_{p}$ in the central spike train can be thought of as standing for a group of spikes $u_{ai}$ from the original spike trains, then there is a residue left behind in $\bar{f}(t)-f(t;\bar{u})$ of $\frac{1}{n}\sum_{u_{ai}<{\bar{u}}_{p}}e^{-|u_{ai}-t|/\tau }\Theta (t,u_{ai},{\bar{u}}_{p})$ where $\Theta (t,u_{ai},{\bar{u}}_{p})$ is a step function which is one for $u_{ai}<t<{\bar{u}}_{p}$ and zero elsewhere.

A better approach is to continue choosing the ${\bar{u}}_{p}$ that minimizes δE, whether this minimum is negative or positive, until the central spike train has a length that matches the average train length in the collection. The quantitative effect this has on central spike trains for the example application is given in the Results section (Sect. 3). In fact, the performance of the central spike train is hardly changed so the choice of one halting criterion over the other appears to be a matter of taste. It depends on whether it is useful to have a central spike train whose spike count matches the average for the collection.

Obviously, from the perspective of the spike train metric, the true average of the spike trains is given by the function average $\bar{f}(t)$. However, this function average is not itself a spike train. The construction described here aims to find the spike train, $\bar{u}$, whose image, $f(t;\bar{u})$, is as close as possible to the function average. This can be seen as a particular instance of the more general question of finding point process prototypes, as explored in [23]. 

Of course, the two functions, $\bar{f}(t)$ and $f(t;\bar{u})$, are not equal. One reason for this is that the set of functions that are in the image of the spike train space

(10) S={f(t;u)|u is a spike train}

will not include $\bar{f}(t)$. This cannot be avoided. However, the aim here is to summarize a collection of responses to repeated presentations of a single stimulus. The function average is not a summary in that its most concise representation is given by the times of all the spikes in $\left\{u_{1},u_{2},\ldots ,u_{n}\right\}$. Since the algorithmic expense of calculating the distance between two spike trains is of the order of the number of spikes [10], this means, for example, that it is as expensive to calculate the distance between a novel spike train **v** and the function average as it is to calculate the distance between **v** and all of the spike trains $\left\{u_{1},u_{2},\ldots ,u_{n}\right\}$ individually. Calculating the distance between **v** and the central spike train is thus *n* times faster.

The second contribution to the difference between $\bar{f}(t)$ and $f(t;\bar{u})$ is the use of the greedy algorithm: $f(t;\bar{u})$ may not actually be the function in S which is closest to $\bar{f}(t)$. Using the greedy algorithm is an efficient way of finding $\bar{u}$, but it is necessary to check that the result is close to the optimal choice of central spike train. This is examined in the Results section (Sect. 3) where a genetic algorithm is used to improve on the greedy algorithm result. It is seen that the improvement is minimal.

## 3 Results

The averaging algorithm has been tested using the very large zebra finch data set collected by the Frederic Theunissen laboratory at UC Berkeley [1] and made available to the Collaborative Research in Computational Neuroscience database. The details of the experiment and of the stimulus set are given in [2,8,26-28]. The data set consists of extracellular recordings from neurons in the auditory pathway of anesthetized zebra finches. Different sound stimuli are used, including a corpus of zebra finch songs. The song responses are considered here. The song corpus generally includes 20 songs. Here, for simplicity, only those data sets with a 20 song corpus and ten trials for each song are used. To make all the spike trains, the same temporal length the first second is used; the length of the stimuli vary, but all are at least one second long. Although the algorithm described here works fine if some empty spike trains are included in the collection to be summarized, for ease of comparison with other methods any cell with empty spike trains is excluded. This gives a total of 183 cells for which a data set of 200 spike trains has been recorded. 

A clustering measure has been used for testing the averaging algorithm. Roughly, the central spike trains are used as a template for clustering by song and the accuracy of this classification is then used as a measure of how well the algorithm performs. Obviously, a test based on distance-based clustering requires the choice of a distance measure between spike trains. Here, the van Rossum metric is used [24]. 

In the van Rossum metric, the distance between two spike trains is calculated by mapping them into the space of functions by filtering and then using the $L^{2}$ metric on that space. The distance between two spike trains **u** and **v** is

(11) $$d(u,v)=\sqrt{\int {\left[f(t;u)-f(t;v)\right]}^{2}\;dt}$$

where f(t;u) and f(t;v) are the filtered spike trains, as before. The van Rossum metric requires a choice of timescale; a choice of the *τ* in the decaying exponential in the kernel, Eq. (3). Here, *τ* is chosen to give the best clustering according to the transmitted information based measure proposed in [25]. It is worth describing this in detail, since a similar measure is used to evaluate the averaging algorithm. 

To estimate the transmitted information measure of metric clustering a confusion matrix *N* is calculated. *N* is a $n_{s}\times n_{s}$ matrix, where $n_{s}$ is the number of stimuli; songs in this case. Starting with all the entries in *N* set to zero, each response is considered in turn. This response is called the test response and its corresponding stimulus is labeled $s_{1}$. The remaining responses are grouped according to their stimulus, giving $n_{s}$ clusters. The distance from the test response to each of these clusters is calculated using a weighted average distance. Therefore, the distance between the test response and a cluster *C* is given by

(12) $$\bar{d}={\left[\frac{1}{\left|C\right|}\sum_{i\in C}d{\left(\text{test response, the response }i\right)}^{z}\right]}^{1/z}$$

where *z* is intended to reduce the effect of outliers, with z=−2 being a typical choice. For each test response, the $n_{s}$ distances are compared and the stimulus corresponding to the smallest distance is noted. Labeling the stimulus corresponding to the nearest cluster as $s_{2}$, one is then added to $N_{s_{1},s_{2}}$.

After each response has been tested in this way, the entries of *N* add up to give the total number of responses. Diagonal elements correspond to responses that were correctly clustered. The transmitted information of the confusion matrix, *h*:

(13) $$\begin{array}{rcl} h & = & \frac{1}{n}\sum_{ij}N_{ij}(\log N_{ij}-\log\sum_{k}N_{kj}\\ & & -\log\sum_{k}N_{ik}+\log\sum_{kl}N_{kl}) \end{array}$$

is a useful measure of the accuracy of clustering indicated by the confusion matrix. The maximum value of the transmitted information *h* is obtained for perfect clustering of $n_{s}$ equally likely stimuli, in which case $h=\log n_{s}$. *h* is the mutual information between the clustering and this perfect clustering [19]. For convenience, a normalized information $\tilde{h}=h/\log n_{s}$ is used.

**Fig. 2:**
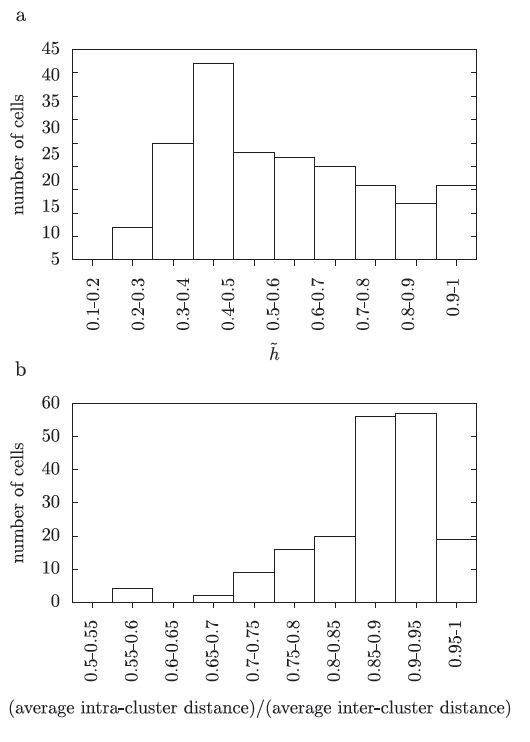
The clustering of the data. (**a**) is a histogram of the optimal $\tilde{h}$ values for the test data in 0.1 bins. (**b**) is a histogram of the ratio between the average intra-cluster and inter-cluster distances. Using the optimal *τ* for each cell, the average distance between responses to the same cell is calculated and divided by the average distance between responses to different cells. This ratio is less than one for all cells. It is plotted here in bins of width 0.05 showing that this ratio is near one for many cells; the average value is 0.87

Here, the *τ* for each cell which gives the highest value of $\tilde{h}$ is used in the metric. This is calculated using the golden section algorithm. It is initialized using the triplet of *τ* values (1 ms,75 ms,150 ms). To give a picture of the challenge addressed by the clustering task, Fig. 2 illustrates the clustering properties of the data by showing histograms of the optimal $\tilde{h}$ values and of the ratio of the distances between responses to the same song and responses to different songs. 

The same *τ* is used in the averaging algorithm. The minimization of the error δE is also performed numerically using a golden section. This is initialized for each iteration of the greedy algorithm with the triplet of ${\bar{u}}_{p}$ values $\left(0,t_{0},1\right)$, where $t_{0}$ is found by evaluating δE at the values 0<rδt<1 for integer *r* and δt=10 ms, and then choosing the value which gives the smallest error.

Although $\tilde{h}$ is intended as a sort of proxy for the information transmitted by the unsupervised clustering of responses using the pairwise distances, it is derived from the supervised procedure described here [25]. This same procedure is used to evaluate the central spike trains. Since the supervised algorithm matches test spike trains against the stimulus-defined clusters, it gives a useful benchmark for a procedure where test spike trains are matched to central spike trains. 

Thus, the central spike trains are evaluated using a transmitted information measure. Again, each response is considered in turn and the remaining responses are clustered by stimulus. In this case, however, the distance between the test response and the clusters is calculated by finding the central spike train for each cluster and measuring the distance between the test response and this central response. Since the test response has been removed from its cluster, it is not used in calculating the central spike train, making this a cross-validation procedure. The confusion matrix and transmitted information are calculated in the usual way.

As a comparison, the transmitted information is also calculated using the same weighted average metric distance defined above, both with z=1, giving the straight-forward average and with z=−2 to underweight outliers. Additionally, the transmitted information is calculated using the function average $\bar{f}(t)$ for each stimulus to cluster the data. Finally, the transmitted information is calculated using the medoid spike train instead of the central one.

**Fig. 3:**
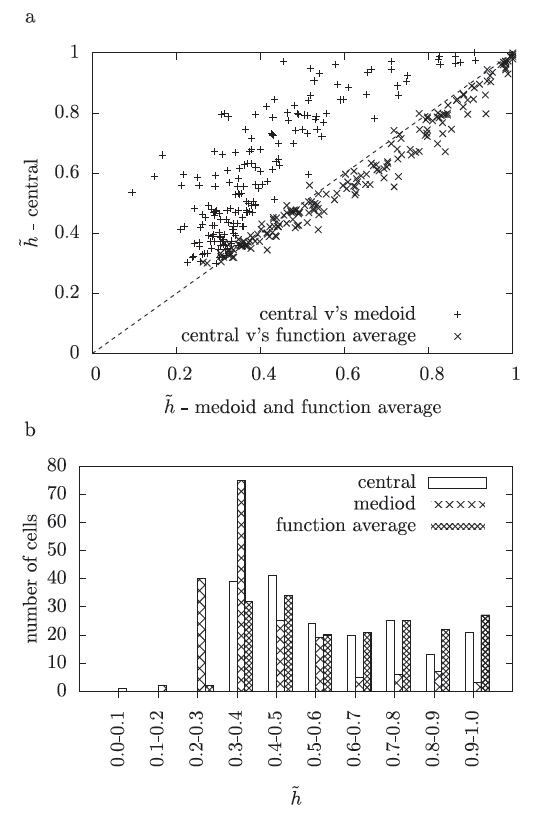
Comparing the transmitted information. In (**a**), for each cell, the value of $\tilde{h}$ for clustering to the central spike train and to the medoid is marked with a +, and for clustering to the central spike train and the function average with a ×. The *dotted line* marks x=y so the points above the line correspond to cells where the central spike train has a higher value for $\tilde{h}$. In (**b**) the histograms show 0.1 bins for the $\tilde{h}$ values for the three clustering methods

The average transmitted information is given in Table 1. This appears to indicate that the central spike train is effective in representing the structure of the spike trains and provides a better exemplar than the medoid spike train. Surprisingly, not only is the transmitted information for the central responses considerably higher than for the medoid responses, it is also higher than the transmitted information calculated using the average distances; both with z=1 and, marginally, with z=−2. The results for the central, medoid, and function average are graphed in Fig. 3. 

**Table 1:** Transmitted information results. In the first column, the transmitted information $\tilde{h}$, averaged over all 183 cells, is given for clustering to the central response (**central**) and to the medoid response (**medoid**). For comparison, the transmitted information is also shown for classification methods which involve all the spike times; the clustering to all the other responses using the z=−2 weighted average distance (**all**z=−2), the clustering to all using the unweighted average distance (**all**z=1) and clustering to the function average (**function**). The second column gives the fraction of cells for which $\tilde{h}$ for the other four clustering methods is larger than $\tilde{h}$ for the **central** clustering. The third column shows the average relative value calculated for each method by dividing the transmitted information for each cell by the transmitted information using the central spike train and averaging, the figure after the ± is the one-sigma variation in this number

	$\tilde{h}$	Better than **central**	Relative to **central**
**Central**	0.60	n/a	n/a
**Medoid**	0.41	0.02	0.70 ± 0.15
**All***z* = −2	0.56	0.16	0.93 ± 0.07
**All***z* = 1	0.52	0.08	0.84 ± 0.12
**Function**	0.62	0.80	1.05 ± 0.07

As described in the Methods section (Sect. 2); the halting criterion for adding spikes to the central spike train specifies that the number of spikes in the central spike train matches the average number of spikes for the collection of spike trains it is summarizing. The effect of using this, rather than the more natural halting criterion based on the error is described in Table 2. It is clear that, in this case at least, the halting criterion does not make a significant difference to performance of the central spike train, though it does, of course, affect the spike count. 

**Table 2:** Halting criteria comparison. In the first column, the transmitted information $\tilde{h}$, averaged over all 183 cells, is given for clustering to the central response (**central**) and to an alternative central response with a different criterion for halting the process of adding spikes (**alternative**). For (**central**), the central response has the same number of spikes as the average, rounding down, for the cluster it summarizes. For (**alternative**) spikes are added to the central response while the δE given in Eq. (9) remains negative. The second column gives the average number of spikes for each. The third column gives the fraction of cells for which (**alternative**) has a $\tilde{h}$ value great than the $\tilde{h}$ value for (**central**), the fourth column gives the average relative value, with the one-sigma variation

	$\tilde{h}$	Spike count	Better than **central**	Relative to **central**
**Central**	0.60	13.0	n/a	n/a
**Alternative**	0.58	10.6	0.45	0.98 ± 0.06

The best clustering comes from using the function average $\bar{f}(t)$. This is unsurprising; as discussed in the Methods section (Sect. 2), the function average represents the average in the function space in which the metric distances are calculated. The aim here is to find a spike train which maps to a point in the subspace of filtered spike trains, S, which is as close as possible to this function average.

**Fig. 4:**
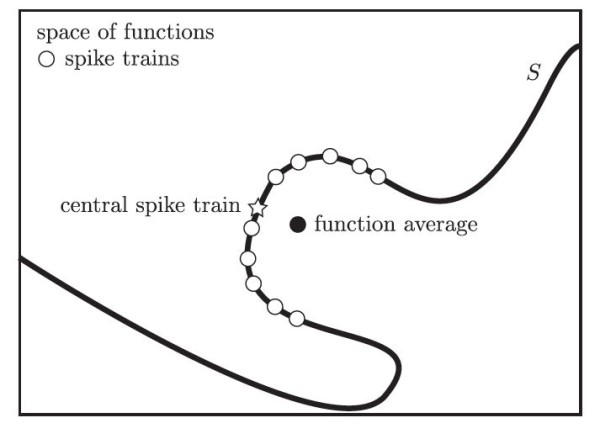
A cartoon representation of the space of functions. Here, the *large rectangle* represents the space of functions, with *S*, the image of the space of spike trains in the space of functions under the filter map represented by the *wavy line*. The spike train functions are marked by *open circles*, the function average by a *closed circle*, and the central spike train function, which is the point on *S* closest to the function average, is marked as a *star*

Because S is a subset of the space of functions, it is inevitable that the image of the central spike train in the space of functions is different from the function average. This situation is represented in Fig. 4. However, it would be a problem if the greedy algorithm was producing functions $f(t,\bar{u})$ that were considerably displaced from the point in S which is closest to $\bar{f}(t)$. To examine this, a genetic algorithm is used to find a new $\bar{u}$ which minimizes the error $E(\bar{u})$ defined in Eq. (7). The initial population of 101 spike trains includes the central spike train calculated using the greedy algorithm. At each step, the best spike train survives and the other 100 are replaced using breeding and mutation, where the probability of a spike train being a parent is determined by its value of ℰ. 

This optimization was performed for the responses to one song chosen at random for each cell. It was found that the distance between the central spike train and the function average after this optimization is 0.967 times what it was before, on average. As a comparison, the distance between the medoid and the function average is, on average, 1.407 times the distance of the central spike train from the function average.

It is possible that there is a middle ground between using the central spike trains and function average to represent a collection of spike trains. One example would be to use a weighted spike train (u,w) with

(14) $$(u,w)\mapsto f\left(t;(u,w)\right)=\sum_{i=1}^{m}w_{i}k(t-u_{i}).$$

This was examined on the example data set by replacing each step of the greedy algorithm with a two-dimensional optimization over spike time and weight using the Nelder–Mead method, initialized using a grid search [14]. However, although this does decrease the error ℰ, it causes only a tiny improvement of the clustering performance. 

Another measure of centrality is the summed distance between a spike train and the spike trains in the collection $\left\{u_{1},u_{2},\ldots ,u_{n}\right\}$. The medoid is chosen as the spike train in the collection that minimizes this distance. As a measure of how central the central spike train is, the summed distance between it and the other spike trains for the song is compared to the summed distance between the medoid and the spike trains. The summed distance for the medoid is, on average, 1.19 times the summed distance for the central spike train. The function average is even more central, its summed distance is on average 0.87 times the summed distance for the central spike train. However, this represents the center of the data in a larger space, the space of functions, rather than the image in the function space of the space of spike trains.

Thus, the medoid is much less central than the central spike train. This may seem surprising since the medoid is chosen as the most central spike train in the collection. However, it is normal in high-dimensional data for the medoid to lie some distance from the center of a data set because the volume around the center is a smaller fraction of the total volume in which the data points fall. For example, for uniform distributed data, the fraction of a unit ball in *D*-dimensions which is within *ϵ* of the center is $\epsilon^{D}$. While it is difficult to associate a dimension with the space of spike trains, the one second spike trains considered here do behave like they belong to a high-dimensional space [9]. 

The idea of ‘central’ is metric dependent and the construction of a central spike train presented here is closely linked to the van Rossum metric; for example, the error function ℰ in Eq. (7) essentially gives the average van Rossum distance between the central spike train and the spike trains in the collection. It might be expected then that the centrality of the central spike train depends on the choice of metric. This has been tested by using the Victor–Purpura edit-distance metric [25] rather than the van Rossum metric to perform the clustering-based evaluation. In the Victor–Pupura metric, there is also a timescale, 2/q, which is analogous to *τ*. Here, *q* is chosen the same way *τ* was chosen: to maximize the transmitted information for clustering using the metric. Table 3 shows the $\tilde{h}$ values. It is found that the central spike train still performs well; it gives $\tilde{h}$ values that are lower than in the van Rossum case, but still considerably higher, on average, than the $\tilde{h}$ values for the Victor–Purpura medoid. 

**Table 3:** Clustering using the Victor–Purpura metric. In the first column, the transmitted information $\tilde{h}$, averaged over all 183 cells, is given for clustering using the Victor–Purpura metric. Here, the central spike train has been calculated in the same way as it has elsewhere, but everything else is calculated using the Victor–Purpura metric; in particular, the medoid is the Victor–Purpura medoid and the clustering performed to calculate the confusion matrix, and hence the transmitted information, depends on the Victor–Purpura distances. As before, **central** gives results for the central spike train, **medoid** for the medoid and (**all**z=−2) and (**all**z=1) use the average weighted and unweighted distances. The function average is not considered in this case since calculating the distance to the function average would involve extending the Victor–Purpura metric to deal with an object of this sort. The second column gives the fraction of cells for which $\tilde{h}$ for the other three clustering methods is larger than $\tilde{h}$ for the **central** clustering, the third column shows the average relative value calculated for each method with the one-sigma variation in this number

	$\tilde{h}$	Better than **central**	Relative to **central**
**Central**	0.53	n/a	n/a
**Medoid**	0.39	0.03	0.74 ± 0.16
**All***z* = −2	0.57	0.83	1.08 ± 0.12
**All***z* = 1	0.53	0.48	0.97 ± 0.17

## 4 Discussion

Although simple and straightforward, the averaging algorithm succeeds extremely well in summarizing the response sets in the data considered here. It is anticipated that this will have numerous practical applications in analyzing sets of electrophysiological responses.

It would be interesting to evaluate the algorithm using different data sets and to measure the extent to which it preserves the internal temporal features of the spike trains. It has been previously noted that averaging over many responses may obscure such features [30]. The aim of this paper is to define a central spike train, an object which can be interpreted as a sort of average, but which is nonetheless a spike train. This is achieved in the sense that the central spike train is a list of spike times, but that does not necessarily mean it shares the less easily-specified properties possessed by real spike trains. For example, real spike trains often have inter-spike interval distributions which are well described by a Gamma distribution or an inverse Gauss distribution [6,7]. However, as a type of average, the central spike train might differ with respect to properties of this sort, precisely because noise has been removed. 

In fact, this is what seems to happen for the data examined in the Results section (Sect. 3). By design, the mean inter-spike interval for the central spike train μ=0.087 s, matches that for the real spike trains, μ=0.085 s. However, the standard deviation of the inter-spike interval length is σ=0.065 s for the central spike train, which is considerably smaller than the value for the real spike trains, σ=0.089 s. Thus, the central spike trains are more regular than the spike trains they summarize. A generative model could be envisaged where the spike times and spike counts of the central spike train are varied to give a collection of spike trains whose statistical properties match those of the original experimental spike trains. Of course, this would involve a fuller investigation of the statistical properties of the central spike train, such as the higher order statistics of the inter-spike intervals [21] and the spike-spike correlation histograms. 

It is hoped that temporal properties crucial to the coding structure of the spike train can be largely preserved in a single exemplar like the central spike train. This is typically the hope when constructing an average; it does not contain all the information present in the original collection of responses, but does include a substantial part of the signal, as opposed to the noise responsible for trial-to-trial variation. In contrast, in the peri-stimulus time histogram the spikes across trials are aggregated into bins. Binning also constitutes an approach to summarizing a collection of trials, but it does so using an object, which does not resemble the original signal. Thus, the peri-stimulus time histogram reduces temporal precision through binning, and the central spike train removes trial-to-trial structure by representing the collection as a single spike train. In each case, information is lost, but it is hoped that this lost information is noise. The peri-stimulus time histogram replaces the original spike trains with something like a rate, the construction here replaces them with the central spike train. In studying coding, this may have the advantage that, as a spike train, the effect of the central spike train on a post-synaptic neuron can be investigated.

One disadvantage of this algorithm is that it requires a value for *τ*, the timescale. Typically, this is chosen so as to optimize the metric clustering and this optimization requires the calculation of the distance matrix for the responses for different values of *τ*. In applications with large data sets where the results are needed rapidly, this might become problematic. Consequently, it would be interesting to consider other methods of choosing *τ* based on easily-accessed properties of the spike trains such as spike number and spike train to spike train variability.

The average is the first moment of a random variable. Often it is useful to also examine quantities such as variance, which are derived from higher moments; for example, it might be interesting to examine whether some types of input produce a noisier output than others. Certainly, it is easy to estimate the variance in the function space, giving a form of the peri-stimulus variance histogram [16]. However, this is a function and it is not clear how to interpret the function variance in terms of spike trains. This would be an interesting topic for further work. Another approach would be to examine the distribution of displacements between the central spike train and the spike trains in the collection, something that has previously been considered using pair-wise displacement in the collection [9]. 

It might also be informative to use the central spike train to average over different neurons rather than over different trials. In this application, deviation from the average would correspond, in part, to aspects of coding which differ from a summed population code.

The choice of the exponential kernel is somewhat arbitrary. However, from the example of the van Rossum metric [11] and of kernel density estimation in statistics [22], it is unlikely that changing the kernel will have a strong effect. One interesting idea might be to use the actual jitter distribution of spike times in the set of responses as a kernel. It would also be interesting to consider the biological basis for the averaging algorithm itself. Perhaps the electrodynamics of neurons can be, in part, interpreted as an averaging algorithm of this sort. Some models of auditory object recognition rely on the use of ‘template’ or ‘memory’ spike trains [12]. Perhaps the central spike train could play a role here. 

## Competing Interests

The authors declare that they have no competing interests.

## Authors’ Contributions

Both authors planned, carried out, and wrote up the research.
